# Drevet Manufacturing Company vs. Liquozone Company

**Published:** 1907-03

**Authors:** Charles Marchand

**Affiliations:** President Drevet Manufacturing Co. New York


### CORRESPONDENCE.

### Drevet Manufacturing Company vs. Liquozone Company.

### Liquozone Barred from Registration in U. S. Patent Office as Unlawfully Interfering with the Trade-mark Glycozone.

Letter from Prof. Marchand, president of the Drevet Manuufacturing Company, giving notice of prosecution in case of infringement.

*Editor Buffalo Medical Journal*:

Sir:—I beg to call your attention to the inclosed legal notice which was mailed to wholesale druggists on February 7, 1907.

I would appreciate your kindness very much if you will publish a notice in the next issue of your Journal, which might read as follows:

"Liquozone" barred from registration in U. S. Patent Office as unlawfully interfering with the trade mark Glycozone.

Notice is hereby given that in a proceeding in the United States Patent Office, which is entitled The Drevet Manufacturing Company vs. The Liquozone Company, the name "Liquozone" was barred from registration in the U. S. Patent Office as unlawfully interfering with the trade-mark Glycozone.

The individual or corporation in any way infringing upon the trade-mark "Glycozone" which is a lawful trade-mark (Glycozone being a thoroughly scientific and legitimate preparation for the treatment of germicidal diseases, etc.,) and duly registered under the new trade-mark law, or selling of any merchandise labeled with any mark or name infringing upon the trade-mark "Glycozone" or in any manner resembling the same, will be prosecuted for damages to the full extent of the law.

Hoping that you will kindly comply with my request so as to help me repair the damage done to our business on account of the confusion between the two names Glycozone and Liquozone, I remain,

Charles Marchand,
President Drevet Manufacturing Co.

New York, February 8, 1907.

